# Mining salt stress-related genes in *Spartina alterniflora* via analyzing co-evolution signal across 365 plant species using phylogenetic profiling

**DOI:** 10.1007/s42994-023-00125-5

**Published:** 2023-12-07

**Authors:** Shang Gao, Shoukun Chen, Maogeng Yang, Jinran Wu, Shihua Chen, Huihui Li

**Affiliations:** 1 State Key Laboratory of Crop Gene Resources and Breeding, Institute of Crop Sciences, Chinese Academy of Agricultural Sciences, Beijing, 100081 China; 2 Nanfan Research Institute, Chinese Academy of Agricultural Sciences, Sanya, 572024 China; 3 Hainan Yazhou Bay Seed Laboratory, Sanya, 572024 China; 4 Key Laboratory of Plant Molecular & Developmental Biology, College of Life Sciences, Yantai University, Yantai, 264005 China; 5 The Institute for Learning Sciences and Teacher Education, Australian Catholic University, Brisbane, QLD 4001 Australia

**Keywords:** Phylogenetic profiling, *Spartina alterniflora*, Salt stress-related gene, Machine learning, Evolutionary information

## Abstract

With the increasing number of sequenced species, phylogenetic profiling (PP) has become a powerful method to predict functional genes based on co-evolutionary information. However, its potential in plant genomics has not yet been fully explored. In this context, we combined the power of machine learning and PP to identify salt stress-related genes in a halophytic grass, *Spartina alterniflora*, using evolutionary information generated from 365 plant species. Our results showed that the genes highly co-evolved with known salt stress-related genes are enriched in biological processes of ion transport, detoxification and metabolic pathways. For ion transport, five identified genes coding two sodium and three potassium transporters were validated to be able to uptake Na^+^. In addition, we identified two orthologs of trichome-related AtR3-MYB genes, *SaCPC1* and *SaCPC2*, which may be involved in salinity responses. Genes co-evolved with *SaCPCs* were enriched in functions related to the circadian rhythm and abiotic stress responses. Overall, this work demonstrates the feasibility of mining salt stress-related genes using evolutionary information, highlighting the potential of PP as a valuable tool for plant functional genomics.

**Supplementary Information:**

The online version contains supplementary material available at 10.1007/s42994-023-00125-5.

## Introduction

Since the start of genomic revolution, thousands of organisms have been sequenced, with many more being sequenced every year. The rapidly increased genomic data of diverse species provide valuable resources for mining unknown genes involved in specific biological processes using comparative genomics approaches (Koonin and Galperin 2003). Of these approaches, phylogenetic profiling (PP) is characterized with the ability to predict functions of less-studied genes in a given species (Tabach et al. 2013a, 2013b). This method compares the presence and absence of genes between species to infer functional linkages in specific biological pathways (Pellegrini et al. 1999). The basic assumption of this approach is that functionally related genes tend to be present or absent from genomes in tandem due to evolutionary constraints.

In the past decades, a series of PP methods have been developed and widely used in human and microbiome studies (Tabach et al. 2013a; Sherill-Rofe et al. 2019). However, very few PP applications have been reported in plants probably due to the relative lack of genomic data. Nowadays, the number of sequenced plant species is reaching nearly 800 (Sun et al. 2021). An enormous project aiming to sequence 10,000 plant and algae genomes is ongoing and scheduled to be completed by 2023 (Cheng et al. 2018). With the sharp increase of released plant genomes, PP offers new opportunities to analyze plant-specific functions that may have evolved in response to environmental pressures.

Salt stress is a major threat to crop yield and quality worldwide (Abobatta 2020). However, halophytes like *Spartina alterniflora* Loisel. (smooth cordgrass) can grow and reproduce in highly saline environments via secreting salt through specialized glands on the leaves and stems (Wang et al. 2006). *S. alterniflora* is phylogenetically close to cereal crops, such as rice, wheat, and maize, making it a valuable model for studying the evolution and function of salt stress-related mechanisms to improve the salt tolerance of crop species. By applying PP to *S. alterniflora*, it is possible to identify novel salt stress-related genes underlying the unique salt-tolerance mechanism of this species.

To identify novel genes associated with a specific biological process, PP methods require a reference list of studied genes involved in the process. This idea is straightforward for model species, in which most of known genes are documented. For less-studied species like *S. alterniflora*, it should be prudent to utilize orthologs of known genes as a direct reference. However, it is important to validate the correspondence between sequence similarity-based homology and evolutionary information used by PP analyses. Such an issue can be addressed by machine learning methods, exemplified by Multilayer Perceptron (MLP) and Random Forest (RF), which have been widely applied in the analyses of biological data (Cheng et al. 2021; Libbrecht and Noble 2015). In addition, it has been proved that co-evolution signals among functionally related genes in different phylogeneitc clades are usually divergent (Stupp et al. 2021). Machine learning models is also able to assess the importance of evolutionary information from different phylogenetic clades, thereby helping to refine the data used in PP analysis.

*S. alterniflora* utilizes salt glands to eliminate detrimental ions, such as sodium, to prevent salt-induced damage. A long-standing hypothesis holds that salt glands and trichomes share a common developmental origin. Yuan et al. (2022) found that two orthologs of trichome-related genes may be involved in salt gland development in *Limonium bicolor*, another extreme halophyte. The developmental mechanism of salt glands remains highly unclear. In contrast, trichome development is well understood in model species like *Arabidopsis thaliana*, involving various transcription factors and complex regulatory networks (Pattanaik et al. 2014; Zhao et al. 2008; Schellmann et al. 2002). Leveraging the predictive capabilities of PP methods, analyzing the orthologs of key trichome-related genes in *S. alterniflora* may help the studies of salt gland development.

*A. thaliana* and *Oryza sativa*, as two most studied model plants, have been reported numerous genes associated with salt stress. Notable representatives include transcription factors and receptor proteins that regulate the abscisic acid (ABA) pathway, the salt overly sensitive (SOS) pathway and ion transport proteins (Van Zelm et al.^2020^). Leveraging the salt stress-related genes reported in *Arabidopsis* and rice, we applied CladePP, an advanced version of PP, to predict genes involving in salt stress response in *S. alterniflora* with evolutionary information generated from a total of 365 plant species. In addition, we also analyzed the phylogenetic profiles of *S. alterniflora* orthologs of *Arabidopsis* genes related to trichome development. In sum, this work validated the feasibility of salt stress-related genes mining using PP, and also established a promising set of candidate genes for future investigation.

## Materials and methods

### Data sources

For the PP analysis, a total of 365 species were selected from diverse clades of the plant kingdom, including green algae, bryophytes, lycophytes, ferns, gymnosperms, and angiosperms. *S. alterniflora*, *A. thaliana*, and *O. sativa* were used as the reference species. Amino acid sequences of all proteins in the 365 species were downloaded from public databases, including Phytozome 13 (Goodstein et al. 2012), Ensembl Plants (Cunningham et al. 2022), and UniProt (The UniProt Consortium 2023), or from species-specific websites (Table S1). The list of sequenced species was extracted from the Published Plant Genomes website (https://plabipd.de, accessed on 1 May 2021).

### Salt stress-related gene curation

A literature review was conducted to establish comprehensive sets of known salt stress-related genes in *A. thaliana* and *O. sativa* (Table S2). The genes were divided into three categories per species: those directly related to salt tolerance (e.g., ion transporters) (At_Salt and Os_Salt); ABA receptor regulators (ABA_RP) and transcription factors involved in regulating the abscisic acid (ABA) pathway (ABA_TF). Orthologs of these genes in *S. alterniflora* were identified using orthoFinder v2.5.5 (Emms and Kelly 2019).

### Generation of normalized phylogenetic profile (NPP) matrices

Three NPP matrices were first built by comparing the proteomes of the reference species (*S. alterniflora*, *A. thaliana*, and *O. sativa*) to the proteomes of the 365 other analyzed species. Proteins of < 40 amino acids in length were filtered out of the reference proteomes. Every protein in each reference proteome was then used as a query to search for the best match among each of the 365 proteomes. This was conducted using the “blastp” function in Diamond v2.1.1 (Buchfink et al. 2015) with default parameters. From this search, we obtained three matrices *BS*, in which *BS*_*i,j*_ was the BLASTP bit-score for the *i*th protein from the reference proteome in the *j*th proteome. When a protein was aligned to itself, the one with a BLASTP score of ≤ 80 were disregarded. To reduce noise, all BLASTP scores below 24.6 (the bit-score value corresponding to an *E*-value of 0.05) were set to 24.6. Reference proteins that did not have a bit-score > 24.6 in at least five other proteomes were discarded from the analysis. To control for differences in protein length, each bit-score *BS*_*i,j*_ was normalized to the bit-score of the reference protein aligned to itself (*BS*_*i, reference*_). Normalization of all values in a matrix yielded an updated matrix, LPP, in which *LPP*_*i,j*_ = *BS*_*i,j*_ / *BS*_*i, reference*._ To control for differences in phylogenetic distance, the values of each column (i.e., species) in *LPP*_*i,j*_ were transformed into *Z*-scores. This led to the final matrix NPP, in which *NPP*_*i,j*_ = (*LPP*_*i,j*_—*μ*_*j*_)/*σ*_*j*_ (where *μ*_*j*_ and *σ*_*j*_ are the mean and standard deviation, respectively, of the *j*th column values).

### Investigating the relationship between sequence-based homology and phylogenetic profiles

#### Data preprocessing

To validate the correspondence between sequence-based homology and evolutionary information, machine learning models were trained based on the NPP matrices of *A. thaliana*, *O. sativa* to predict the *S. alterniflora* orthologs of literature curated salt stress-related genes. Since the sample size was sufficiently large, we approximated the population mean and population standard deviation with the sample mean (*μ*) and sample standard deviation (*σ*), respectively. Following the 3-sigma rule, we considered observations outside the range of *μ* ± 3σ for each variable as outliers, and we replaced them with null values. Additionally, variables and samples with more than 20% of missing values were discarded. Linear interpolation was conducted to impute missing values in the matrices. Common variables across the three species were selected for following model training. The *S. alterniflora* orthologs of the curated genes were used as labels.

#### Training machine learning models

The training datasets exhibited imbalance in the sample size of positive and negative cases. To address this issue, we employed the Cluster-Centroids method, a clustering-based undersampling technique, to balance the positive and negative samples in the training set. This method involved clustering the majority class samples using the *K*-means algorithm and subsequently reducing the majority class samples by replacing each cluster’s samples with their center. The implementation of the Cluster-Centroids method was achieved using the “ClusterCentroids” class from the Python package imbalanced-learn” (Lemaître et al. 2017).

The MLP algorithm, a classical artificial neural network model, was first chosen for classification. We used the “MLPClassifier” class from the scikit-learn package (Pedregosa et al. 2011) to implement the MLP algorithm. It consists of three hidden layers with 180, 90, and 40 neurons, respectively, and each layer fully connected to the adjacent layers. All of the hidden layers are activated by ReLU nonlinearity function. Adam optimizer was used to update the parameters of MLP models. RF, a classical ensemble learning algorithm, was another classification method used in this study. It builds multiple decision trees and aggregates their predictions through voting to make final classifications. The "RandomForestClassifier" class from the scikit-learn package was used to implement the RF algorithm. Grid search was employed to optimize the hyperparameters of the two models with the “GridSearchCV” class from the scikit-learn package. According to the grid search results, the initial learning rate λ = 0.0001 and the L2 regularization coefficient *α* = 0.1 were applied for all the MLP models. For the RF models, the number of estimators and the max depth was, respectively, set to 55 and 15. Accuracy, precision, recall, and F1-score were used to evaluate the classification performance.

### Mining salt stress-related genes in *S. alterniflora* using phylogenetic profiling

To identify novel salt stress-related genes on phylogenetic clades-wise level, CladePP (Sherill-Rofe et al. 2019) was applied to the three NPP matrices containing all species and the clades which contains more than ten species. These clades were Chlorophyta (29 species), Poales (47), Rosales (30), Fabales (25), Malpoghiales (12), Malvales (32), Brassicales (17), Sapindales (10), Ericales (13), Lamiales (21), and Solanales (16). To account for local co-evolution within clades, CladePP can perform hierarchical clustering on subsets of columns in the matrix. For example, to examine co-evolution within angiosperms, only the columns corresponding to angiosperm species would be clustered. CladePP uses Ward’s method for hierarchical clustering, which minimizes the total within-cluster variance. The output of CladePP is a list of hclust objects, with one for each clade of interest. To identify genes that co-evolved with a set of query genes within a clade, CladePP computes a maximal ratio score (MRS) for each gene based on its phylogenetic profile similarity with the query gene(s). MRSs range from 0 to 1, with values closer to 1, indicating that a gene is highly similar to the query gene(s). The top 1% genes with highest MRS were classified as candidate salt stress-related genes in *S. alterniflora*.

### Gene ontology (GO) term enrichment analysis

GO term enrichment analyses were performed for the co-evolved genes. Genome-wide gene annotations based on TAIR identifiers were obtained from the R package ‘org.At.tair.db’ (Carlson 2019) and used as the reference background for annotations in *A. thaliana*. The reference backgrounds used for *S. alterniflora* and *O. sativa* were constructed with an in-house pipeline. The enrichGO function in the R package ‘clusterProfiler’ (v4.8.1) (Wu et al. 2021) was used to perform hypergeometric tests to identify over-represented GO terms among the co-evolved genes.

### Data processing and visualization

All data used in this study were processed and visualized in R 4.2.1 (R Core Team 2013) using the R packages ‘tidyverse’ (Wickham et al. 2019), ‘ggplot2’ (Wickham 2011), ‘pheatmap’ (Kolde 2017), and ‘UpSetR’ (Conway et al. 2017).

### Validation of predicted genes coding ion transporter

For the construction of yeast expression vectors, the CDSs of all ion transporter genes were amplified from *S. alterniflora* seedlings cDNA and cloned into the p416-GDP vector. The *AxT3K* yeast strain (*Δena1::HIS3::ena4, Δnha1::LEU2, Δnhx1::KanMX4*) was used to transform the recombinant plasmids using LiAc methods and was selected on SD/-Ura synthetic dropout medium and subjected to growth on AP medium with different concentrations of NaCl. The yeast cells were plated on the AP medium in a tenfold serial dilution with OD_600_ values in the range of 0.6 to 0.6 × 10^–4^.

### Gene expression analysis in *S. alterniflora*

Expression levels of trichome-related orthologs in *S. alterniflora* were analyzed with RNA-sequencing data (unpublished data). The dataset consisted of samples from the roots, stems, leaves, and seeds. The leaves had been treated with a water control or with 300 or 600 mM NaCl for 48 h. Differentially expressed genes (DEGs) between tissues and treatment groups were determined using the R package ‘edgeR’ (v3.42.4) (Robinson et al. 2010). The thresholds for DEG classification were *p*.adj < 0.05 and |log_2_(fold change)|> 1. In terms of the validation of *SaCPCs* expression pattern, the method for the rhythmic treatment of *S. alterniflora* is as follows: the *S. alterniflora* at the flowering stage is cultivated under the condition of 100 mM NaCl, where 8:00 is the beginning of the day and 20:00 is the beginning of the night, and the leaves are sampled every 4 h. Each treatment was conducted with three biological replicates. For the isolation of total RNA and the synthesis of first-strand cDNA were carried out according to the instructions of the products (TIANGEN, Beijing). For real-time quantitative reverse transcription PCR (RT-qPCR), 15 μL reaction system containing 7.5 μL SYBR mix (Accurate Biology, Changsha), 0.5 μL cDNA (200 ng/μL), 0.75 μL (10 pmol/μL) each of forward and reverse primers (Sangon Biotech, Shanghai), and 5.5 μL ddH_2_O. The reaction conditions were 50 °C for 2 min and 95 °C  for 10 min at the pre-denaturation stage, followed by 40 cycles of 95 °C for 15 s, 60 °C for 1 min at the PCR stage, and 95 °C for 15 s, 60 °C for 1 min, and 95 °C for 15 s at the melt curve stage. Data acquisition was carried out by a LightCycler® 96 system (Roche, Switzerland), and the relative expression levels of the target genes were calculated using 2^–ΔΔCt^ method and normalized to the *SaGAPDH*.

## Results

### Evolutionary trajectories of salt stress-related genes in green plants

**Fig. 1:**
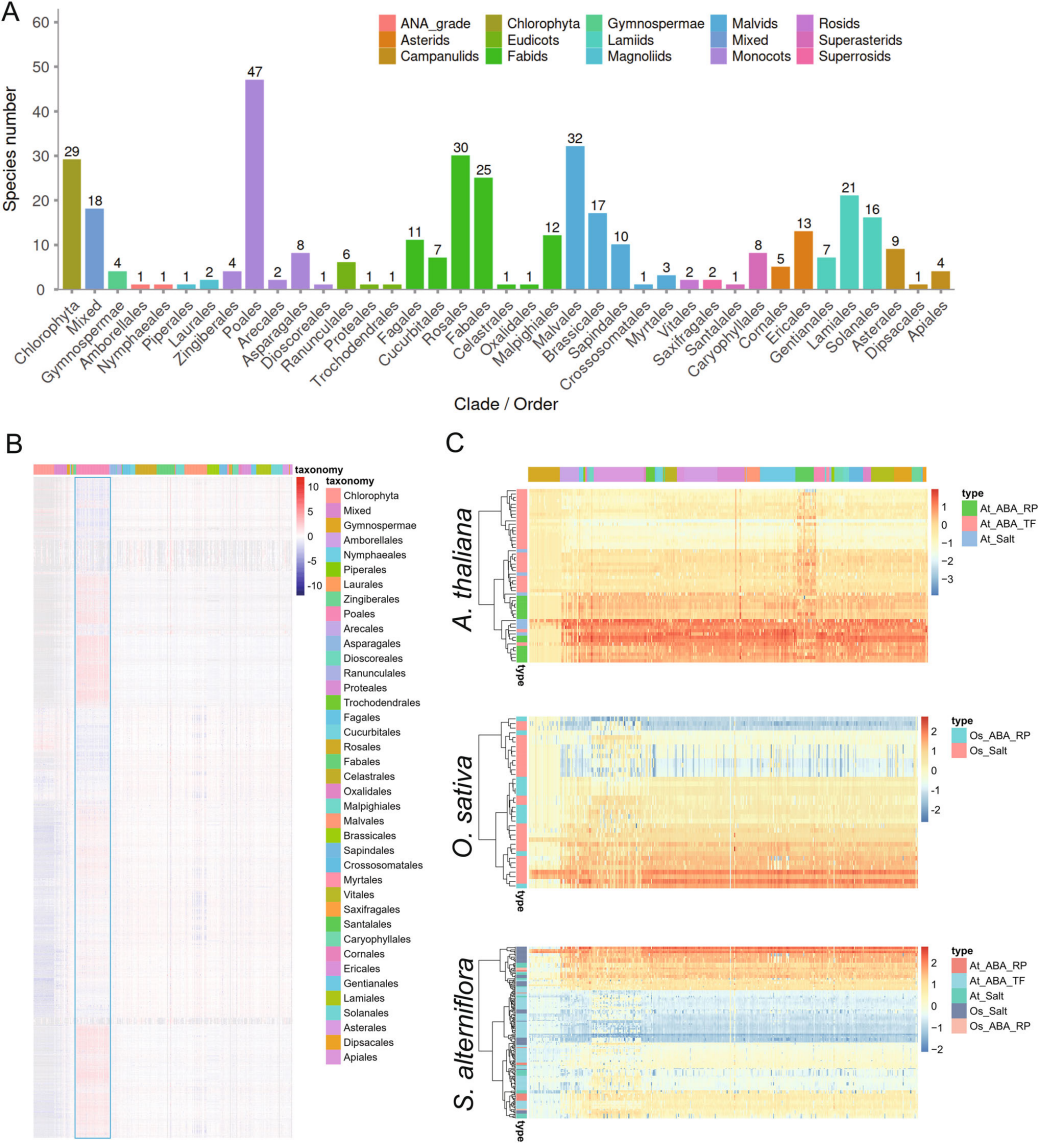
Evolutionary trajectories of genes related to salt-stress in selected plant species. **A** Distribution of the 365 analyzed plant species among each of the 40 subgroups. The phylogenetic profiling matrices were normalized based on the number of species in each subgroup to form the normalized phylogenetic profiling (NPP) matrices. **B** NPP matrix for all protein-coding *S. alterniflora* genes after hierarchical clustering and dendrogram leaf order optimization. Each row represents the NPP for a single gene across 365 eukaryotes, ordered by phylogenetic distance from basal angiosperms. The relative degree of conservation between each *S. alterniflora* protein and its closest ortholog in each species (column) is indicated by color. Zero (white) corresponds to average conservation of an ortholog relative to *S. alterniflora*; negative values (blue) correspond to lower-than-average conservation; and positive values (red) correspond to higher-than-average conservation. Conservation values are expressed as Z-scores. The coloring along the top of the graph indicates the order/clade to which the corresponding column belongs. **C** NPP patterns for known salt stress-related genes in *Arabidopsis thaliana* (upper) and *Oryza sativa* (middle) and for orthologs of those genes in *S. alterniflora* (lower)

We sought to establish the evolutionary trajectories, i.e., phylogenetic profiles, of salt stress-related genes in green plants. To accomplish this, we first constructed three NPP matrices for three reference species (*A. thaliana, O. sativa,* and *S. alterniflora*) across a total of 365 green plant species, which were divided into 40 subgroups at the clade/order level (Fig. 1A). Each row of an NPP matrix represented a protein in the reference species and each column represented one of the 364 non-reference species (Fig. 1B). The matrices were rendered as heatmaps to visualize the highest sequence similarity score of each reference protein in each other species. As expected, because *S. alterniflora* belongs to the family Poaceae, the *S. alterniflora* genes generally had higher sequence similarity scores in members of the Poaceae clade than in members of other clades (Fig. 1B). Similar results were observed in the NPP matrixes of *A. thaliana* and *O. sativa* (Fig. S1).

Known salt stress-related genes were manually curated for *A. thaliana* and *O. sativa*, and all orthologs of these genes were identified in *S. alterniflora* to form a set of putative salt stress-related genes in the halophyte (Table S2). We divided the salt-tolerant genes into three functional types: (1) general salt stress-related genes, such as the Na^+^/H^+^ antiporter gene *SOS1* and the sodium transporter gene *HKT* (At_salt and Os_salt); (2) transcription factors in the ABA pathway, such as *AtAREB1/2* (ABA_TF); and (3) regulatory elements of ABA receptors, including members of the *PYL* family (ABA_RP). Notably, the genes related to salt stress clearly clustered together in the NPP matrices. The evolutionary characteristics of functionally similar genes tended to be similar; in fact, salt stress-related genes further clustered together by functional type (Fig. 1C). Such findings demonstrated that the phylogenetic profiles of each gene were consistent with potential functional interactions across multiple branches of the evolutionary tree.

### Exploring the relationship between phylogenetic profiles and sequence similarity-based homology using machine learning

**Fig. 2:**
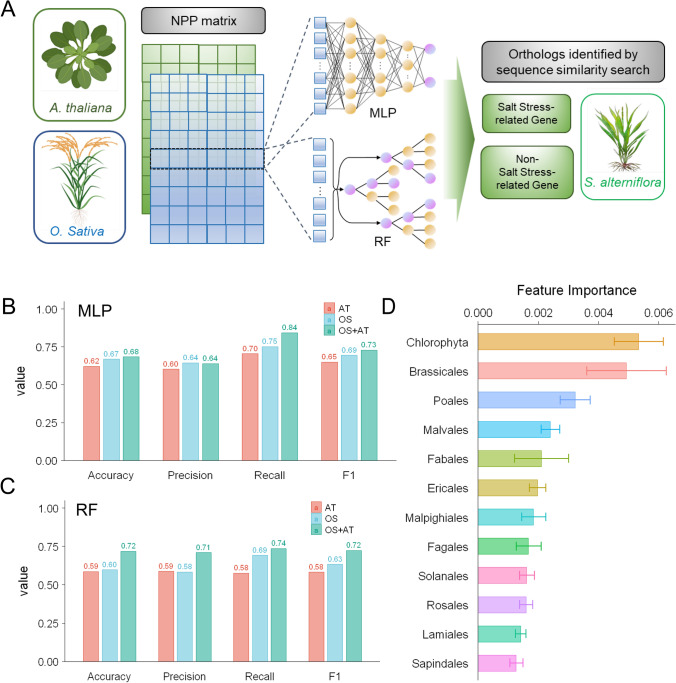
Salt stress-related genes are predictable across species by machine learning using evolutionary information. **A** The NPP matrices of rice (OS) and *Arabidopsis* (AT), and the combined dataset (OS + AT) were used to train multi-layer perceptron (MLP) and random forest (RF) classifiers to investigate the relationship between genes’ phylogenetic profiles and their sequence-similarity-based homology. **B** Prediction performance of the MLP models on the *S. alterniflora* dataset. **C** Prediction performance of the RF models on the *S. alterniflora* dataset. **D** Feature importance of the joint RF model for rice and *Arabidopsis*. All species are grouped by clade, and clades containing no less than ten species are depicted. Error bars represent standard errors within each clade

The *S. alterniflora* orthologs of salt stress-related genes in *A. thaliana* and *O. sativa* were acquired using sequence-similarity search. Different from sequence-similarity-based homology, phylogenetic profiling detects functional relationship between genes using their evolutionary information among the tree of life. It is of worth to explore the relationship between salt stress-related genes’ homology and their phylogenetic profiles in green plants. Therefore, we used machine learning methods to investigate the relationship across species (Fig. 2A). We first trained MLP and RF models to detect the prediction ability of phylogenetic profiles for salt stress-related genes in *A. thaliana* and *O. sativa*. Both types of models demonstrated high predictive performance on datasets from *A. thaliana* and *O. sativa*, or their combination, with all prediction accuracy exceeding 80%. The RF model achieved a prediction accuracy of up to 91.6% on *A. thaliana* (Fig. S2). Next, we trained models to predict *S. alterniflora* orthologs, and the single-species (AT or OS) MLP models outperformed the single-species RF models in terms of all performance statistics. The combined (AT + OS) MLP model also outperformed the RF model in terms of F1-score (Fig. 2C, D). This might due to the stronger capacity of MLP to capture nonlinear information across species. Overall, the prediction results of these models show that there is a corresponding relationship between phylogenetic profiles and sequence-based homology in cross-species prediction.

The feature importance of the combined RF model suggests that the clade phylogenetically closer to the target species contributed more to the classification accuracy (Fig. 2E). Brassicales and Poales, the parent clade of *A. thaliana* and *O. sativa*, are two of the top three contributors. The significant contribution from the Chlorophyta lineage likely indicates its substantial influence on identifying negative class. Such results imply that, while mining target functional genes from phylogenetic profiles, attention should be paid not only to global evolutionary information but also to specific branches. Therefore, we conducted CladePP analysis on the NPP matrix of *S. alterniflora* to further detect salt stress-related genes.

### CladePP revealed novel salt-tolerant genes in *S. alterniflora*

**Fig. 3:**
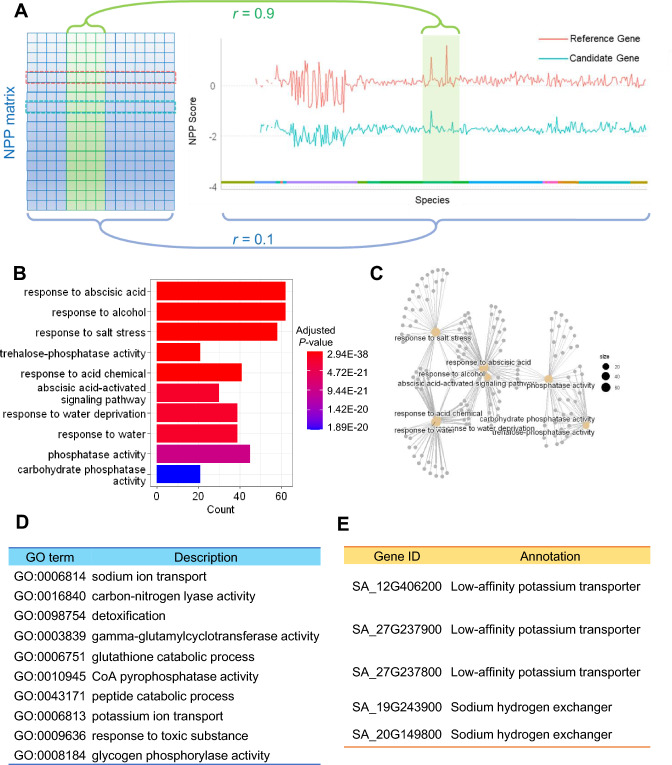
Gene Ontology (GO) terms enriched among CladePP predicted genes, which co-evolved with salt stress-related genes in *S. alterniflora*. **A** Diagram of the global and local co-evolution signal detected by CladePP. The left panel is the complete NPP matrix, and the green part indicates a specific clade. The line graph shows the co-evolution of a reference gene (pink) and a candidate gene (cyan). The X-axis represents different species. **B** GO term enrichment among putative salt stress-related genes in *S. alterniflora*. **C** Network of genes and the top ten highly enriched GO terms in *S. alterniflora.* **D** GO terms enriched among genes co-evolved with putative salt stress-related genes in *S. alterniflora*. **E** IDs of candidate genes annotated as ion transporters

**Fig. 4:**
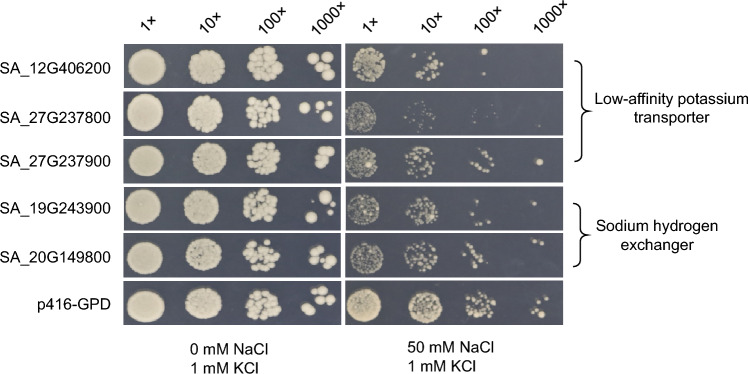
Functional validation of the ion transporter-coding genes predicted by CladePP. Growth of yeast strain *AxT3K* cells expressing the five ion transporters and the vector control (p416-GPD) under different concentrations of NaCl. All plates were incubated at 30 °C and photographed after 3–5 days

To find local co-evolutionary signals in certain phylogenetic clades, we performed CladePP on the NPP matrix of *S. alterniflora*. CladePP is an advanced version of phylogenetic profiling (PP) which designed to detect co-evolution signal between reference genes and candidate genes within specific evolutionary clades, especially when co-evolution pattern is difficult to detect on a global level (Fig. 3A). A total of 351 orthologs of the curated salt stress-related genes were identified in *S. alterniflora*. GO term enrichment analysis confirmed that these orthologs were primarily involved in abiotic stress. These were annotated in GO terms related to ABA, salt stress, and drought responses (Fig. 3B, C). For the CladePP results, the top 1% highly co-evolved genes were enriched in GO terms, including sodium ion transport (GO:0006814), potassium ion transport (GO:0006813), and detoxification and metabolic pathways (Fig. 3D). Five co-evolved genes were annotated as ion transporters: *SA_12G406200*, *SA_27G237900*, and *SA_27G237800*, which are predicted to encode low-affinity potassium transporters; and *SA_19G243900* and *SA_20G149800*, which are predicted to encode sodium hydrogen exchangers (Fig. 3E). We further used *AxT3K*, a yeast strain that lacks Na^+^ transport activity and is salt sensitive, to detect the Na^+^ transport of these five candidate genes. As a result, salt stress (50 mM NaCl) inhibited the growth of cells expressing these five genes and they were weaker than the control p416-GDP, indicating that they have the ability to uptake Na^+^ and mediate the salt stress response (Fig. 4). These results preliminarily validated the use of CladePP analysis to identify functionally related genes in *S. alterniflora*.

### CPC orthologs were present in *S. alterniflora* but not in other grass species

**Fig. 5:**
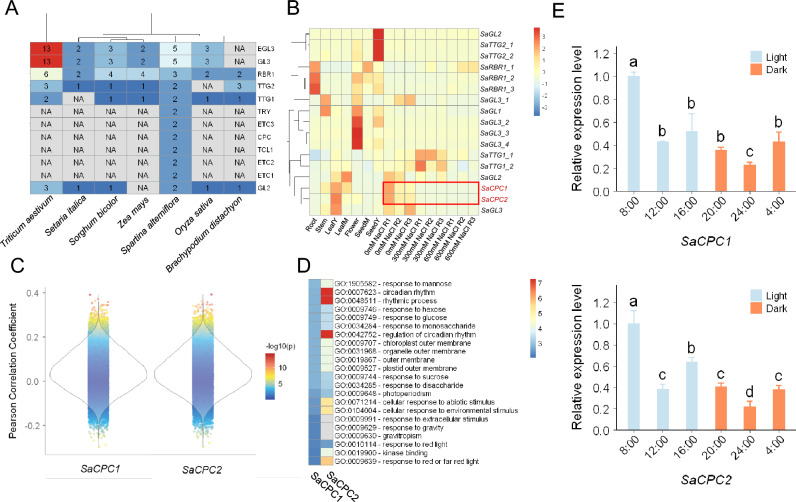
Retention of R3-MYB orthologs in *S. alterniflora* compared to other Poaceae species. **A** Number of orthologs of *Arabidopsis* trichome development genes among selected Poaceae species. NA, no homolog detected in the indicated species. **B** Expression levels of *Arabidopsis* trichome development orthologs in *S. alterniflora.* Expression levels in each sample are represented as Z-scores. Each column represents a specific tissue or salt treatment condition. Samples ending in “Y” and “M” indicate young and mature tissues, respectively; samples ending in “RX” (where X is 1–3) are experimental replicates. **C** Gene expression correlation analysis for two *SaCPCs*. **D** Enriched GO terms for the 1% of most highly correlated genes with *SaCPC1* and *SaCPC2*. Color indicates the –log_10_-transformed *P* value from GO enrichment analysis. **E** Analysis of the expression patterns of two *SaCPC* genes changing with rhythm. The horizontal axis represents different time points, 8:00–20:00 is light time, 20:00–8:00 is dark time; the vertical axis represents relative expression levels. Values are means ± SD. Different letters represent significant differences at *P* < 0.05 (Duncan’s multiple range test)

*S. alterniflora* develops salt glands, but lacks trichomes. This is consistent with the long-standing hypothesis that halophyte salt glands and trichomes have a shared origin. We therefore studied orthologs of known trichome-related genes in major cereal crop species and *S. alterniflora*. Notably, orthologs of R3-MYB family members, including CPC, TRY, ETC1, ETC2, ETC3, and TCL1, were present only in *S. alterniflora* in the Poaceae clade (Fig. 5A). The two orthologs were designated as *SaCPC1* and *SaCPC2*. Using an unpublished transcriptomic dataset for *S. alterniflora*, we assessed the expression levels of these trichome-related orthologs in multiple tissues and in response to treatment with two concentrations of NaCl. The *SaCPC*s were found to be most highly expressed in the young leaves of plants grown without salt stress. Both genes were significantly downregulated in response to treatment with either 300 or 600 mM/L NaCl (Fig. 5B). It is not unreasonable to infer that *SaCPCs* regulate salinity response in the *S. alterniflora* through salt gland development.

We next further investigated the functions of genes that co-evolved with *SaCPC*s. Correlation analyses of phylogenetic profiles were performed between the *SaCPC* genes and every other gene in the *S. alterniflora* genome to evaluate the most co-evolved genes with *SaCPC1* and *SaCPC2*. The correlation coefficients showed a normal distribution (Fig. 5C). GO enrichment analyses were conducted with the top 1% genes that were most highly co-evolved with each *SaCPC*. GO terms related to abiotic stress responses (GO: 0071214), circadian rhythm (GO: 0007623, GO: 0048511), and sugar metabolite response were enriched (Fig. 5D). We also conduct the correlation and enrichment analyses for AtR3-MYB members. Their functions in *Arabidopsis* are related to trichome formation and differentiation (Fig. S3), in contrast to the enrichment results in *S. alterniflora*. Such findings reflect the functional divergence in *CPC* gene between *S. alterniflora* and *A. thaliana*. It is of interest that circadian rhythm GO terms were detected in the co-evolved genes of *SaCPCs*. We conducted RT-qPCR assays to detect the expression patterns of *SaCPCs* along time (Fig. 5E). The results indicated that the expression of *SaCPC1* and *SaCPC2* displayed significant rhythmic changes during day and night.

## Discussion

Phylogenetic profiling (PP) analyses are used to predict functionally related genes through identification of protein families that are consistently present or absent together across the tree of life (Pellegrini et al. 1999). PP has already been widely applied to study evolution in microbial communities and humans. For example, a study in human identified small RNA pathway genes using PP (Tabach et al. 2013a). Gene involved in essential cellular processes like DNA repair (e.g., BRCA1) and energy metabolism (e.g., PGC-1α) were also investigated with their conserved co-evolution patterns across a wide range of species (Sherill-Rofe et al. 2019). Few studies to date have applied PP in plants. However, the ever-increasing number of publicly available plant genome sequences offers new opportunities to generate valuable insights with this method. An advanced version of PP, CladePP, can detect both local and global co-evolutionary signals from different clades of the tree of life, offering both high sensitivity and specificity. In the present study, we successfully implemented CladePP to a collection of 365 plant genomes and identified a range of genes related to salt stress response in *S. alterniflora*.

Analyses of the genes identified by CladePP revealed several detoxification and metabolism-related enzymes and transporters, such as glutathione S-transferase, cytochrome P450, and ABC transporters. These genes may be involved in relieving oxidative stress and ion balance disorders caused by high-salt stress. CladePP also identified five co-evolved ion transporter genes: two sodium–hydrogen exchangers and three potassium ion transporters. Sodium–hydrogen exchangers are transmembrane proteins that can exchange intracellular H^+^ with extracellular Na^+^, thereby reducing intracellular Na^+^ concentrations and maintaining the intracellular pH balance (Blumwald 2000). Low-affinity potassium transporters are channel proteins capable of transporting K^+^ at high concentrations, which can improve selective uptake of potassium and sodium by plants under high-salt conditions (Maathuis et al. 1997). By exchanging H^+^ with Na^+^ or transporting K^+^ at high concentrations, the ion transporter genes can help plants cope with high-salt stress and avoid ion toxicity or deficiency. Complementary assays in yeast indicated that these five salt ion transporters are involved in the salt response and mediated Na^+^ transport, which further demonstrated the advantage of being able to identify core genes through CladePP.

In addition to salt stress-related genes, we also analyzed orthologs of trichome development genes, which may be related to salt gland development in *S. alterniflora*. Clade-specific analysis of known trichome development orthologs demonstrated that two R3-MYB genes (*CPC*s) were present in an *S. alterniflora*-specific evolutionary trajectory among Poaceae species. Notably, the *CPC* orthologs in *S. alterniflora* were highly expressed in untreated young leaves, but downregulated in response to salt treatment. In *Arabidopsis*, CPC acts as a negative regulator of trichome development, preventing trichome formation by inhibiting the MBW complex. In *S. alterniflora*, *SaCPC* may act as a positive regulator of salt gland development, activating salt gland differentiation by promoting formation of the MBW complex. This hypothesis is consistent with our finding that *SaTTG1* (a component of the MBW complex) was upregulated in response to salt treatment, and with the likely involvement of an *L. bicolor* TTG ortholog in salt gland differentiation and development (Yuan et al. 2022). Thus, the results of this study supported the hypothesis that trichomes and salt glands have shared origins.

Analysis of genes that co-evolved with *SaCPC*s revealed functional enrichment of annotations related to the circadian rhythm and abiotic stress responses. These co-evolved genes may form a complex network with *SaCPCs* to coordinate *S. alterniflora* responses to salt stress and photoperiod changes. Circadian rhythm is an important regulator of plant physiological and metabolic processes and is one of the key mechanisms by which plants adapt to environmental changes (Harmer 2009). Previous studies have shown that the circadian rhythm can affect plant sensitivity to salt stress (Dodd et al. 2005; Park et al. 2016). RT-qPCR results demonstrated that the expression patterns of the two *SaCPC* genes change with rhythm, and we therefore hypothesize that *SaCPC* regulates *S. alterniflora* adaptations to salt stress via the circadian rhythm.

In summary, the candidate genes identified by CladePP contribute to molecular understanding of salt-stress resistance mechanisms in *S. alterniflora*, and this method offers new avenue for exploration of abiotic stress tolerance in crop species.

### Supplementary Information

Below is the link to the electronic supplementary material.

Supplementary file1 Fig. S1 Normalized phylogenetic profiles (NPPs) of all *Arabidopsis thaliana* (A) and *Oryza sativa* (B) protein coding genes after hierarchical clustering and dendrogram leaf order optimization (PPTX 1249 KB)

Supplementary file2 Fig. S2 Prediction performance of the MLP (A) and RF (B) models on test datasets of *A. thaliana* (AT), *O. sativa* (OS) or the combined (AT + OS) (PPTX 91 KB)

Supplementary file3 Fig. S3 GO term enrichment results of trichome development-related R3-MYB members in *A. thaliana* (PPTX 260 KB)

Supplementary file4 (XLSX 27 KB)

## Data Availability

The datasets generated and analyzed during this study are available at https://huggingface.co/datasets/AIBreeding/PhylogeneticProfiling/tree/main or from the corresponding author on reasonable request.
