# Supplementary figures and images for: Mining salt stress-related genes in Spartina alterniflora via analyzing co-evolution signal across 365 plant species using phylogenetic profiling
Source: aBIOTECH. 2023 Dec 7;4(4):291–302. doi: 10.1007/s42994-023-00125-5 (PMC10721760; doi:10.1007/s42994-023-00125-5)

## Slide 1
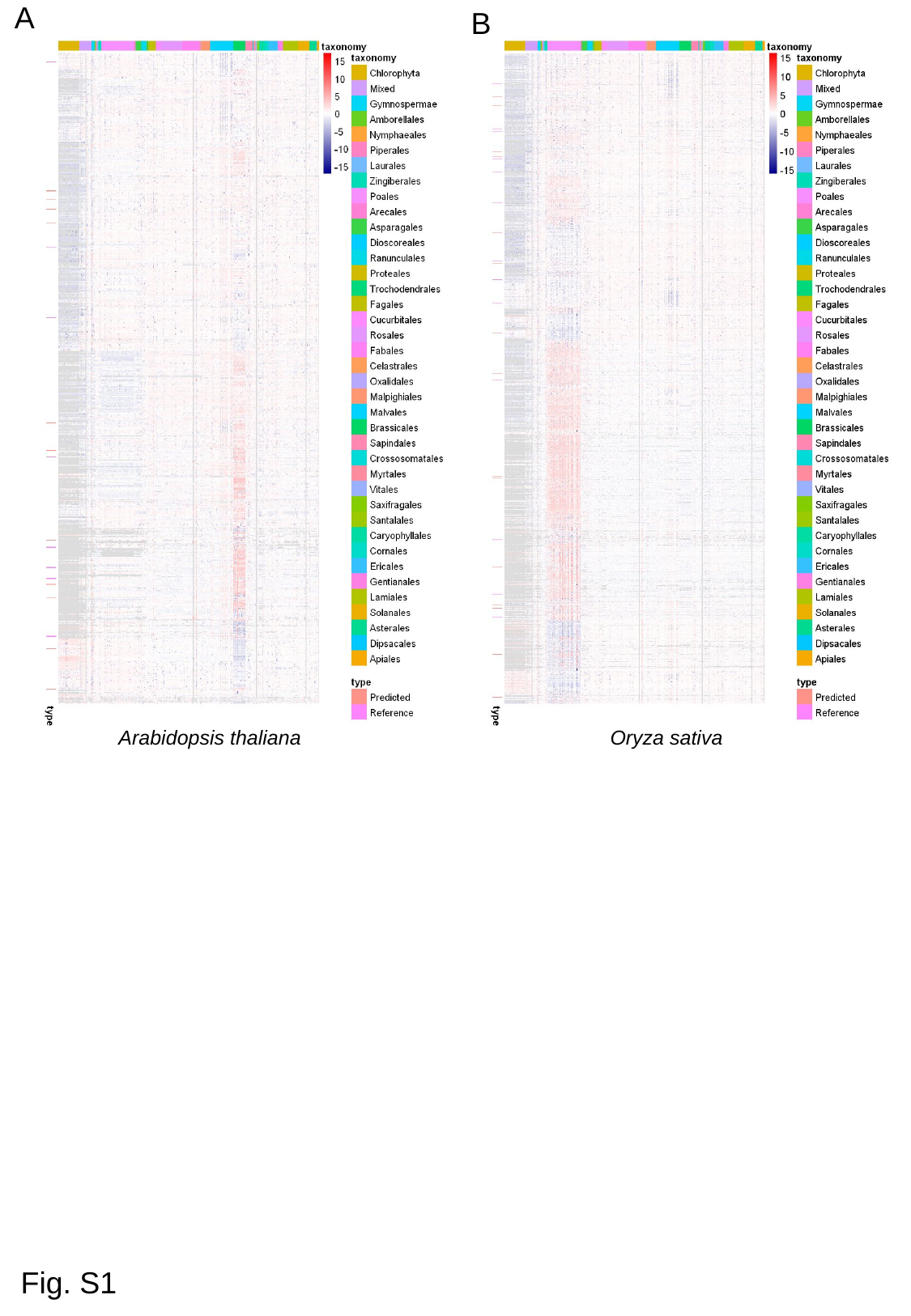

A
B
Arabidopsis thaliana
Oryza sativa
Fig. S1

Supplement: Supplementary file 1 — Supplementary file1 Fig. S1 Normalized phylogenetic profiles (NPPs) of all Arabidopsis thaliana (A) and Oryza sativa (B) protein coding genes after hierarchical clustering and dendrogram leaf order optimization (PPTX 1249 KB) [file 42994_2023_125_MOESM1_ESM.pptx]

## Slide 1
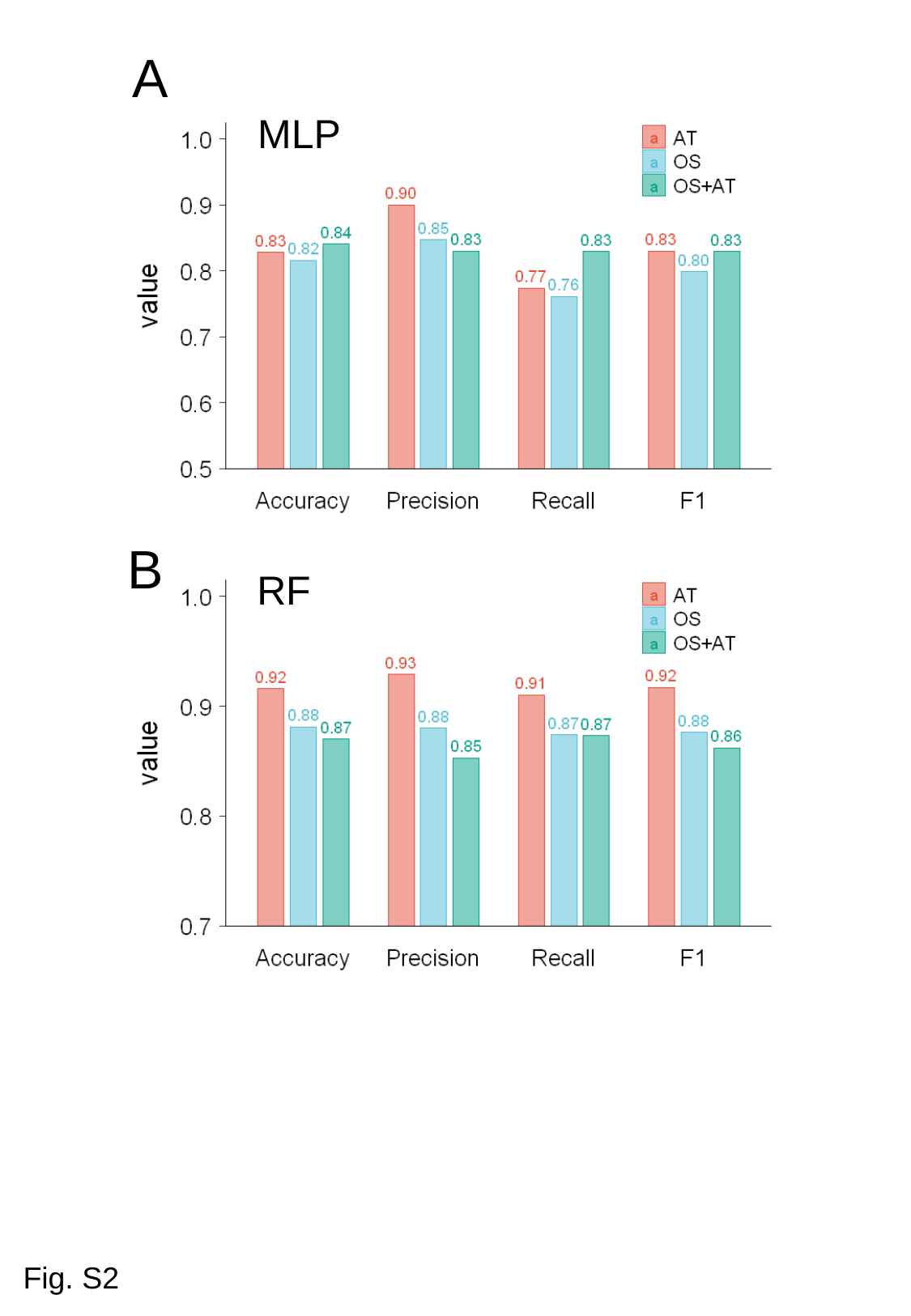

A
MLP
B
RF
Fig. S2

Supplement: Supplementary file 2 — Supplementary file2 Fig. S2 Prediction performance of the MLP (A) and RF (B) models on test datasets of A. thaliana (AT), O. sativa (OS) or the combined (AT + OS) (PPTX 91 KB) [file 42994_2023_125_MOESM2_ESM.pptx]

## Slide 1
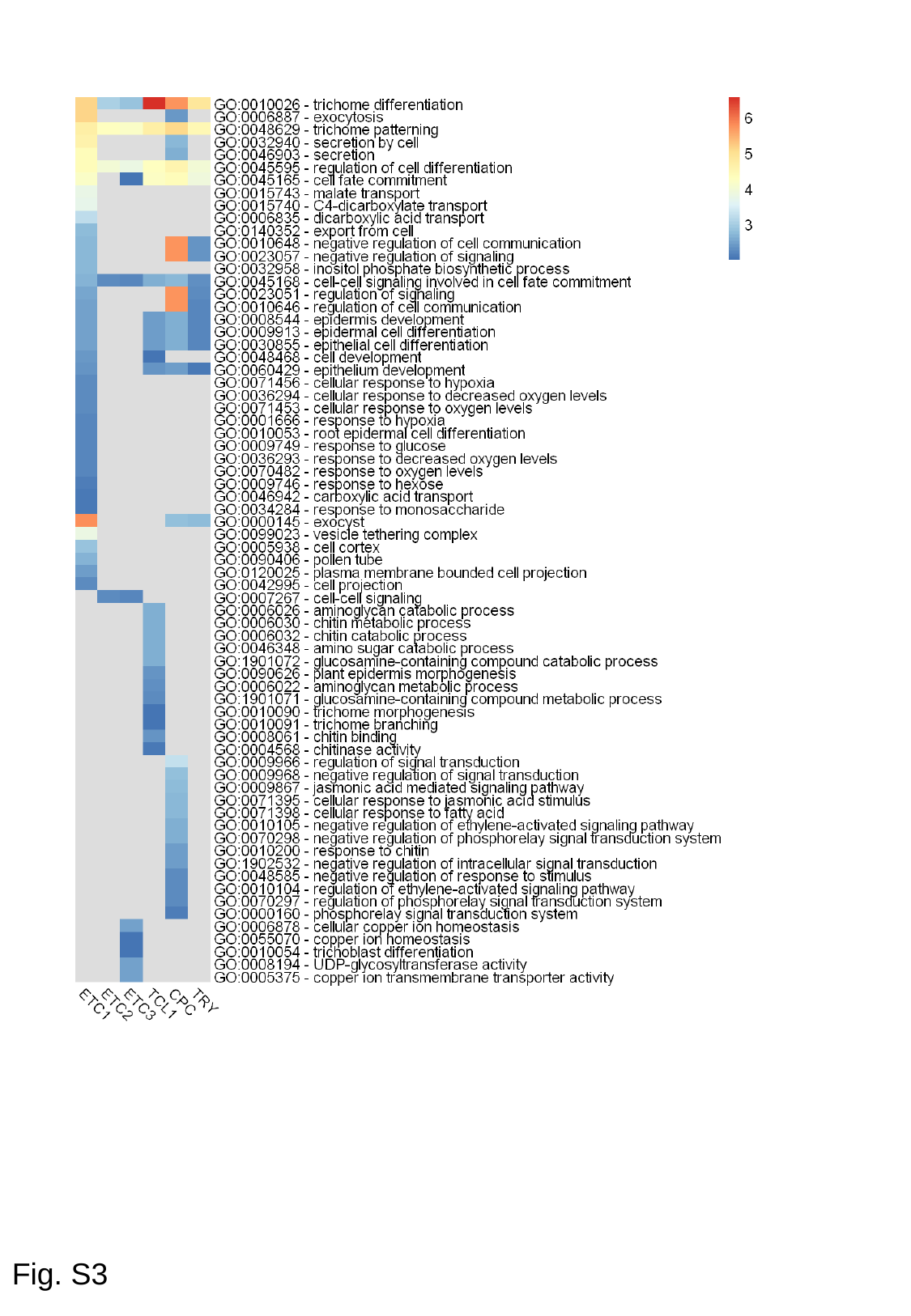

Fig. S3

Supplement: Supplementary file 3 — Supplementary file3 Fig. S3 GO term enrichment results of trichome development-related R3-MYB members in A. thaliana (PPTX 260 KB) [file 42994_2023_125_MOESM3_ESM.pptx]
